# A delta-doped quantum well system with additional modulation doping

**DOI:** 10.1186/1556-276X-6-139

**Published:** 2011-02-14

**Authors:** Dong-Sheng Luo, Li-Hung Lin, Yi-Chun Su, Yi-Ting Wang, Zai Fong Peng, Shun-Tsung Lo, Kuang Yao Chen, Yuan-Huei Chang, Jau-Yang Wu, Yiping Lin, Sheng-Di Lin, Jeng-Chung Chen, Chun-Feng Huang, Chi-Te Liang

**Affiliations:** 1 Department of Physics, National Tsinghwa University, Hsinchu, 300, Taiwan; 2 Department of Electrophysics, National Chiayi University, Chiayi, 600, Taiwan; 3 Department of Physics, National Taiwan University, Taipei, 106, Taiwan; 4 Department of Electronics Engineering, National Chiao Tung University, Hsinchu, 300, Taiwan; 5 National Measurement Laboratory, Centre for Measurement Standards, Industrial Technology Research Institute, Hsinchu, 300, Taiwan

## Abstract

A delta-doped quantum well with additional modulation doping may have potential applications. Utilizing such a hybrid system, it is possible to experimentally realize an extremely high two-dimensional electron gas (2DEG) density without suffering inter-electronic-subband scattering. In this article, the authors report on transport measurements on a delta-doped quantum well system with extra modulation doping. We have observed a 0-10 direct insulator-quantum Hall (I-QH) transition where the numbers 0 and 10 correspond to the insulator and Landau level filling factor ν = 10 QH state, respectively. *In situ *titled-magnetic field measurements reveal that the observed direct I-QH transition depends on the magnetic component perpendicular to the quantum well, and the electron system within this structure is 2D in nature. Furthermore, transport measurements on the 2DEG of this study show that carrier density, resistance and mobility are approximately temperature (*T*)-independent over a wide range of *T*. Such results could be an advantage for applications in *T*-insensitive devices.

## Introduction

Advances in growth technology have made it possible to introduce dopants which are confined in a single atomic layer [1]. Such a technique, termed delta-doping, can be used to prepare structures which are of great potential applications. For example, many novel structures based on delta-doped structures [2-10] can be experimentally realized using very simple fabrication techniques. It is found that delta-doped quantum wells may suffer from surface depletion and carrier freeze-out, which compromise their performances, thereby limiting their potential applications. To this end, a delta-doped quantum well with additional modulation doping can be useful. The modulation doping provides extra electrons so as to avoid carrier freeze-out. On the other hand, it preserves the advantages of a delta-doped quantum well structure, such as an appreciable radiative recombination rate between the two-dimensional electron gas (2DEG) and the photo-generated holes [9], and an extremely high 2DEG density, suitable for high-power field effect transistor [8]. It is worth mentioning that doped quantum wells with additional modulation doping [11-16] have already been used to study the insulator-quantum Hall (I-QH) transition [17-23], a very fundamental issue in the fields of phase transition and Landau quantization. In order to fully realize its potential as a building block of future devices, it is highly desirable to obtain thorough understanding of the basic properties of a delta-doped quantum well with additional modulation doping. In this article, extensive resistance measurements on such a structure are described. At low temperatures (0.3 K ≤ *T *≤ 4.2 K), the authors have observed a low-field direct I-QH transition. *In situ *tilted-field experiments demonstrate that the observed direct I-QH transition only depends on the magnetic field component applied perpendicular to the quantum well, and thus the electron system within our device is 2D in nature. Resistivity, carrier density, and hence mobility of the device developed are all weakly temperature dependent. These results may be useful for simplifying circuitry design for low-temperature amplifiers, and devices for space technology and satellite communications since extensive, costly and time-consuming tests both at room temperature and at low temperatures may not be required.

## Experimental details

The sample that we used in these experiments was grown by molecular beam epitaxy (MBE). The layer sequence was grown on a semi-insulating (SI) GaAs (100) substrate as follows: 500 nm GaAs, 80 nm Al_0.33_Ga_0.67_As, 5 nm GaAs, Si delta-doping with a density of 5 × 10^11 ^cm^-2^, 15 nm GaAs, 20 nm undoped Al_0.33_Ga_0.67_As, 40 nm Al_0.33_Ga_0.67_As layer with a Si-doping density of 10^18 ^cm^-3^, and 10 nm GaAs cap layer. It is found that electrical contacts to a delta-doped quantum well with the same doping concentration do not show Ohmic behaviour at *T *< 30 K. Therefore, additional modulation doping is introduced in order to provide extra carriers so as to avoid this unwanted effect. As shown later, the carrier density of the 2DEG is indeed higher than the delta-doping concentration. Moreover, the electrical contacts to the 2DEG all show Ohmic behaviour over the whole temperature range (0.3 K ≤ *T *≤ 290 K). Both results demonstrate the usefulness of additional modulation doping. The sample was processed into a Hall bar geometry using standard optical lithography. The sample studied in this study is different from that reported in Ref. [14] but was cut from the same wafer. Low-temperature magnetotransport measurements were performed in a He^3 ^cryostat equipped with an *in situ *rotating insert. Transport measurements over a wide range of temperature were performed in a closed-cycle system equipped with a water-cooled electric magnet.

## Results

**Figure 1:**
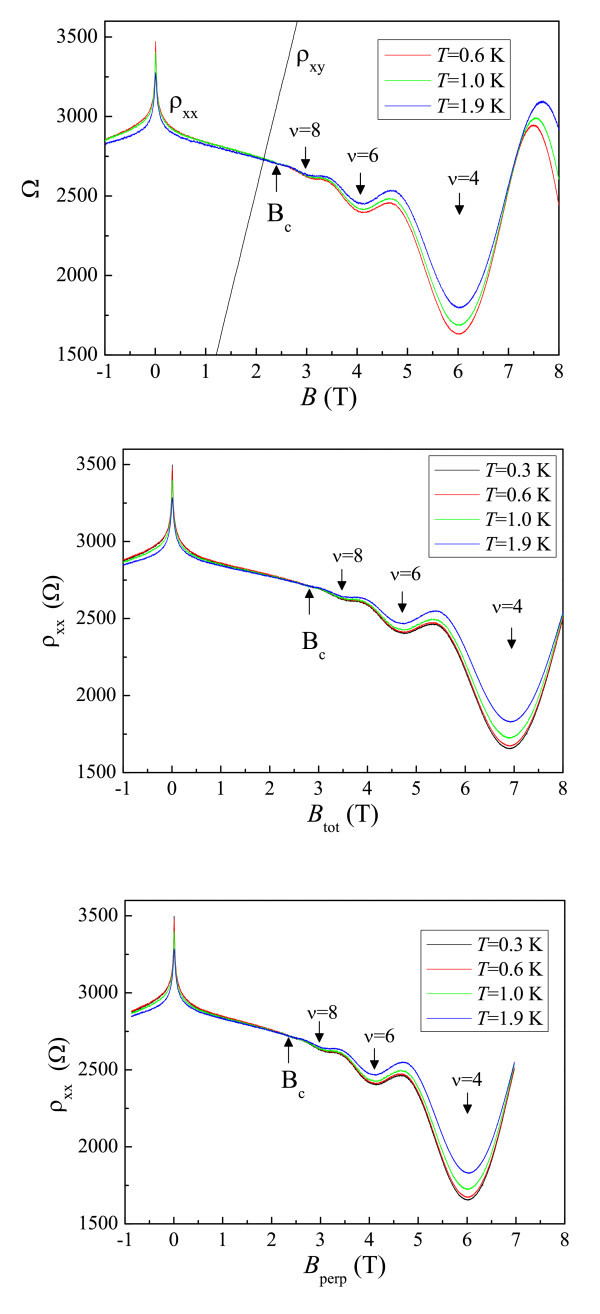
**Four-terminal magnetoresistance measurements:(a)** Longitudinal resistivity ρ_*xx *_measurements as a function of magnetic field ρ_*xx*_(*B*) at various temperatures. Hall resistivity ρ_*xy *_as a function of *B *at *T *= 1.9 K is shown. **(b) **Longitudinal resistivity measurements as a function of total magnetic field ρ_*xx*_(*B*_tot_) at various temperatures. **(c) **Longitudinal resistivity measurements as a function of the perpendicular component of the applied magnetic fieldρ_*xx*_(*B*_perp_) at various temperatures.

In the system developed in this study, ionized Si dopants confined in a layer of nanoscale can serve as nano-scatterers close to the 2DEG. Figure 1a shows longitudinal and Hall resistivity measurements at various temperatures when the magnetic field is applied perpendicular to the plane of the 2DEG. Minima in ρ_*xx *_corresponding to Landau level filling factors ν = 8, 6 and 4 are observed. On the other hand, ρ_*xy *_is linear at around ν = 8 and 6, and shows only a step-like structure, not a quantized Hall plateau at around ν = 4. We can see that at the crossing field *B*_c_, approximately 2.4 T, where the corresponding filling factor is about 10, ρ_*xx *_is approximately *T*-independent. Near the crossing field, ρ_*xx *_is close to ρ_*xy*_. Therefore, we observe a low-field direct I-QH transition, consistent with existing theory and experimental results [13-16,18-22]. In order to further study this effect, the sample was tilted *in situ *so that the angle between the applied *B *and growth direction is 28.5°. Figure 1b shows ρ_*xx *_and ρ_*xy *_as a function of total magnetic field which is applied perpendicular to the 2DEG plane at various temperatures. The ν = 4 QH-like state is now shifted to a higher field of *B *approximately, 7 T. Similarly, the crossing field is shifted to a higher field of approximately, 2.9 T. The authors now re-plot the data as a function of perpendicular component of the total magnetic field, as shown in Figure 1c. It can be seen that both crossing field and the minimum in ρ_*xx *_corresponding to the ν = 4 QH-like state are now the same as those shown in Figure 1a. The results therefore demonstrate that the electron system are indeed 2D in nature since all the features only depend on the *B *component perpendicular to the growth direction. Furthermore, the corresponding approximately *T*-independent point in ρ_*xx *_at the crossing field is the same, despite an in-plane magnetic field of approximately 1.4 T being introduced in our tilted-field measurements.

**Figure 2:**
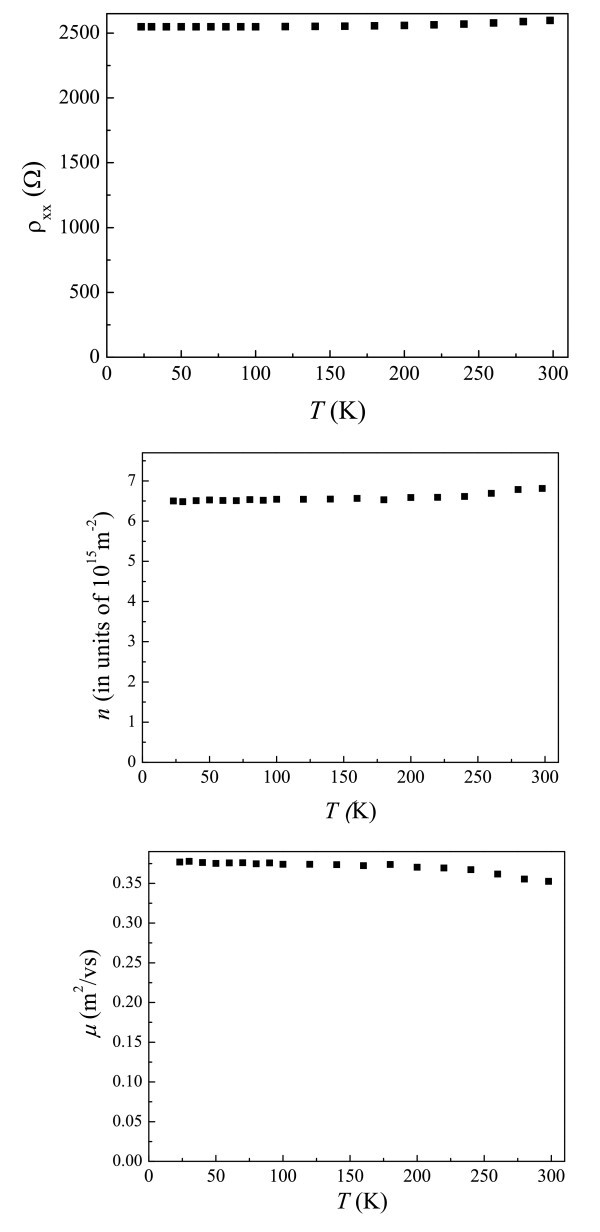
**Electrical measurements over a wide range of temperature:(a)** Resistivity as a function of temperature ρ_*xx*_*(T)*, **(b)** carrier density as a function of temperature *n(T)*, and **(c)** mobility *as a function of *temperature μ*(T)*.

As mentioned earlier, it is highly desirable to obtain a thorough understanding of the basic properties of our system so as to fully realize its potential in electronic and optoelectronic devices. Figure 2a shows resistivity measurements as a function of *T *over a wide range of temperature. Interestingly, ρ_*xx *_is almost *T*-independent from room temperature down to 23 K. To understand why ρ_*xx *_at *B *= 0 is insensitive to the temperature, the *T*-dependence of *n *is investigated, and *μ *is obtained using ρ_*xx *_= 1/*neμ *at zero magnetic field, as shown in Figure 2b, c. The carrier concentration does not decrease too much, and thus the 2DEG does not suffer from the carrier freeze-out at low temperatures because of the extra modulation doping. While *μ *increases with decreasing *T *in most 2DEG because of the reduced electron-phonon scattering, it can bee seen from Figure 2c that *μ *saturates and remains at approximately 0.37 m^2^/v/s from *T *= 230 K. For a 2DEG in the delta-doped quantum well, with decreasing *T*, it shall be considered that the enhancement of the multiple scattering may decrease the mobility and thus compensate the reduced electron-phonon scattering effect [6,7]. Therefore, we can design the devices insensitive to *T *by using the delta-doped quantum well with the extra modulation doping. For example, when designing a circuit for a low-temperature amplifier, such as the one used for space technology and satellite communications, one needs to perform a test at room temperature (RT) first. When cooling down the amplifier, its characteristics can be significantly different since the resistance of the device based on HEMT structure may be a lot lower than that at RT [24]. Therefore substantial variation in the circuitry design based on the RT test is required. Since the ρ_*xx*_, *n *and *μ *of our structure are almost *T*-independent over a wide range of temperature, a RT test may be sufficient.

Both the strong and weak localization effects can compensate the reduced electron-phonon effect with decreasing *T*. To clarify the dominant mechanism leading to the compensation in this study, it is noted that the direct I-QH transition inconsistent with the global phase diagram of the quantum Hall effect reveals the absence of the strong localization [17,18]. The magneto-oscillations following the semiclassical Shubnilkov-de Haas formula when *B *< 6T also indicates that the strong localization is not significant near *B *= 0 [14,23]. Therefore, the weak localization effect should be responsible for the enhancement of the multiple scattering, compensating for the reduced electron-phonon effect [25].

## Conclusions

In summary, electrical measurements of a delta-doped single quantum well with additional modulation doping have been presented. A direct I-QH transition in such a structure has been observed. *In situ *tilted-field measurements demonstrate that the observed 0-10 transition only depends on the magnetic field component applied perpendicular to the quantum well, and therefore the electron system within the sample studied is 2D in nature. Neither carrier freezeout nor second electronic subband at a high density of 6.5 × 10^15 ^m^-2 ^is observed in the system proposed. Transport measurements over a wide range of temperature reveal that ρ_*xx*_, *n *and *μ *all show very weak *T *dependencies. These results could be useful for devices which can maintain their characteristics over a wide range of temperature. Our results could also be useful for circuit design for low-temperature amplification, and devices for space technology and satellite communications.

## Abbreviations

2DEG: two-dimensional electron gas; I-QH: insulator-quantum Hall; MBE: molecular beam epitaxy; RT: room temperature; SI: semi-insulating; *T*: temperature.

## Competing interests

The authors declare that they have no competing interests.

## Authors' contributions

DSL, LHL, YTW and ZFP performed the low-temperature tilted-field measurements. YCS, STL, and KYC performed the measurements over a wide range of temperature. YHC started the project. CFH and CTL drafted the manuscript. YL and JCC coordinated the measurements. JYW processed the sample. SDL grew the MBE wafer. All authors read and approved the final manuscript.
